# Facial affect recognition and exit examination performance in medical students: a prospective exploratory study

**DOI:** 10.1186/s12909-014-0245-6

**Published:** 2014-11-28

**Authors:** Tessa C Roos, Dana JH Niehaus, Jukka M Leppänen, Johan Ras, Karen J Cloete, Esmè Jordaan, Liezl Koen

**Affiliations:** Department of Psychiatry, Faculty of Medicine and Health Sciences, Stellenbosch University, PO Box 19063, Tygerberg Cape Town, 7505 South Africa; Human Information Processing Laboratory, School of Social Sciences and Humanities, University of Tampere, Tampere, FIN-33014 Finland; Biostatistics unit, Medical Research Council, Parow, South Africa; Statistics and Population Studies Department, University of the Western Cape, Cape Town, South Africa

**Keywords:** Emotional intelligence, Facial affect recognition, Examination performance, Undergraduate medical students, Gender

## Abstract

**Background:**

Facial affect recognition (FAR) abilities underpin emotional intelligence (EI). The latter is suggested to predict academic success and to be important for clinician-patient interaction. It is therefore of interest to investigate the possible association between FAR and academic performance in undergraduate medical students.

**Methods:**

We assessed the association between the ability to recognize emotions through facial expression and exit examination performance, a measure of clinical proficiency, in undergraduate medical students stratified by gender at a South African tertiary institution using a prospective descriptive design. Data on the perception of facial expressions and exit examination marks were obtained from 144 (61%) females and 93 (39%) males with a mean age of 24.1 ± 1.6 years. Facial affect recognition measures on the Hexagon and Animation tasks were individually correlated with academic performance indicators using Pearson correlation.

**Results:**

The perceptual discrimination of anger was associated with improved performance in anaesthetics (*r* = .24; *p* = .004) and urology (*r* = .24; *p* = .001), while the recognition of happiness was associated with decreased performance in obstetrics (*r* = −.21, *p* = .002). Gender was an effect modifier in the relationship between perceptual discrimination of anger and urology performance (*p* = .03), with a strong positive relationship for males, but a non-significant relationship for females.

**Conclusion:**

There was no overall correlation between FAR and overall academic performance or with gender. However, subject (specialty) specific findings with recognition of specific emotions and with gender as effect modifier poses interesting questions about EI and FAR and prompts further research into FAR as a useful tool. Being an objective test and offering a more focused assessment makes FAR worthy of further application.

## Background

There has been increased interest in attributes besides cognitive ability that predict academic success in medical students, with particular emphasis on the complex concept of emotional intelligence (EI) [1,2]. There is also ongoing debate on training medical students in EI skills [2-5]. Facial affect recognition offers a potential building block for EI, and it may form a piece of the puzzle.

Emotional intelligence has been linked with clinician-patient interaction, patient satisfaction and teamwork [2,6-8]. EI has also been linked to empathy [2]. Although book knowledge is essential, the ultimate delivery of care to a patient and their family involves far more than this. As medicine has moved away from the paternalistic attitude of the past towards one that prioritises patient rights and preferences, this issue has gained importance.

Despite this, the concept and application of EI remains controversial. What the concept is, if it is a trait or an ability, whether it is psychometrically sound or not are a few of the questions asked [9]. Emotional intelligence has broad definitions; one being the ability of an individual to monitor and regulate emotions, to promote emotional and intellectual growth through competence in perceiving, using, facilitating, and understanding emotions [10].

What then, is facial affect recognition (FAR)? As the face is often used to express and perceive distinct emotions non-verbally, the ability to read emotions from facial expression or affect is considered to be one of the most important components of emotional intelligence [11,12]. Recognition of emotion through facial expressions relative to other kinds of expressive information has also been found to be more accurate [13,14]. Females are claimed to have a greater ability to recognize facial affect than males [15-17] although this too is contested [9].

One of the most commonly accepted theoretical models of EI relies on emotion perception i.e. recognition of facial affect, as a lower order branch upon which higher order branches such as emotion management depend [18]. Moving away from broad assessments of EI, which are controversial, and looking at possible underlying constructs, could contribute to this interesting field. Many tests of EI are self-report measures while facial affect recognition tests are more suited to objective testing. This makes them ideal for use in an undergraduate population where using EI as part of the training is being debated.

The association between emotional intelligence and academic performance is also debated [9]. For example, some studies show weak associations between EI and academic performance [19], whereas others show no association [20]. Interestingly, specific entities of EI (e.g. intrapersonal, stress management and adaptability subscales of the BarOn Emotional Quotient Inventory [21] have been shown to predict academic success [22-24]. Despite this, studies assessing the association between the ability to recognize emotions through facial affect, and academic performance in undergraduate medical students are lacking.

In this report, we specifically assessed the association between the ability to recognize emotions through facial affect and exit examination performance, a measure of clinical proficiency, in undergraduate medical students stratified by gender at a South African tertiary institution. Specific objectives included 1) to assess associations between blended and dynamic facial affect recognition measures; 2) to assess associations between FAR measures and academic performance measures in males and females; and 3) to measure the strength of significant associations between FAR and academic performance measures. We hypothesized that there would be an association between FAR and academic performance measures and that gender would be an effect modifier in the association.

## Methods

### Study design and setting

We conducted a prospective, descriptive study at the Servier Student Training Center at Stikland Hospital, South Africa from 2008 to 2010.

### Sample

Participants (n = 237) consisted of final-year (late-rotation) undergraduate medical students from Stellenbosch University, Faculty of Medicine and Health Sciences. An independent social scientist, who was not linked to the student evaluation or academic program, facilitated the recruitment procedure.

### Data collection

We collected demographic data on the students’ age and gender and their examination scores for all final year courses, see Table 1. The examination format included written, oral, objective structured clinical examination (OSCE) and objective structured practical examination (OSPE) marks combined with the class mark. Each subject utilizes a different assessment format. For example; in psychiatry there is an oral examination, in urology a written examination paper and three clinical case-based orals are required, obstetrics and gynecology (O&G) utilizes eight OSCE and four (OSPE) stations and anaesthesiology uses an OSCE design. Academic performance was defined as the final mark (%) obtained in each of the clinical subjects.

**Table 1:** **Mean percentage (SD) exit examination scores of medical students at Stellenbosch University (n = 237)**

	**Mean%**	**SD**
Ear Nose and Throat	67.2	10.2
Oncology	64.2	7.2
Urology	63.8	7.0
Obstetrics	63.6	8.9
Surgery	63.4	7.1
Family medicine	63.2	6.5
Internal medicine	62.8	7.3
Anesthesiology	62.6	10.6
Pediatrics	62.4	7.1
Orthopedics	62.1	8.8
Psychiatry	61.4	9.2
Mean performance	62.6	8.9

SD: (standard deviation).

Assessment of facial affect recognition was completed using two measures of perceptual discrimination of facial recognition namely the computerized Hexagon and Animation tasks described below. All medical students rotated through psychiatry during the final year of their medical degree. All students were approached for participation and no one declined. Data were pooled for the study period. Demographic and academic performance data were available for two hundred and fifteen students that rotated through psychiatry in early 2008 prior to the start of the FAR testing and after the conclusion of FAR testing late in 2010. This data was used to assess possible recruitment bias i.e. differences in student profiles over time.

### Facial affect recognition tasks

Participants (n = 237) completed two computerized tasks assessing FAR; the Hexagon and Animation tasks. We selected these tasks as they are tried and tested stimulus sets, with proven sensitivity to individual variations [25,26]. The Animation and Hexagon tasks were predicted to assess partially distinguishable aspects of emotion processing. The Animation task reflects purely perceptual discrimination of facial expressions, without the need for labeling the expressions displayed on the face [25]. The Hexagon task, in turn, is cognitively more demanding because this task requires the ability to label emotional expressions, an important component of emotional intelligence [26].

Previous studies suggest that a dissociable neural network may be involved in the tasks requiring mere perceptual discrimination versus tasks requiring labeling, with the latter being associated with heightened activity in limbic-prefrontal systems [27]. Our general prediction was that each task assessed a different factor underlying emotional intelligence, and hence was expected to be associated with academic performance.

The Hexagon task is from the established FEEST test with validated Ekman Faces. It uses the images of human faces from the Ekman and Friesen (1976) series. These images were originally tested in the 1970s on US born college students to establish the initial Ekman 60 Faces test. This test has strong validity and has been used extensively in research. The Hexagon test was then developed from this test and strong correlation was found between the two. Norms for the Hexagon test exist for gender, age and IQ but it has no norms in an African context [26].

In the first (Hexagon) task [26], participants were asked to recognize happy, sad, angry, fearful, disgusted, and surprised facial expressions from morphed images. Morphed or blended images refer to a widely used image manipulation technique in which; pictures of two prototypical facial expressions are "mixed" or "blended" by computerized image morphing to create a blend of the two (e.g., a face that shows 90% of a prototypical happy expression and 10% of a prototypical angry expression) [26]. The participants viewed the images on a computer screen and reported their responses using a computer keyboard. The percentage of correct responses out of 12 images was calculated for each emotion category and used as a dependent variable in the analyses.

In the second (Animation) task [25], participants were shown animations of happy, sad and angry facial expressions, and were asked to use a slide bar to play the animation forwards and backwards until they detected the offset point for each facial expression (i.e., the point at which the happy, sad or angry expression changed to a neutral face) [25]. The frame (0–100) on which the subject determined that there was no longer an emotion expressed on the face was automatically saved and used as a dependent variable in the analyses. Participants viewed a total of 8 animations per category, and the average offset points were calculated separately for happy, and sad expressions.

Students completed the tests in isolation from other students and no conferring was possible. Prior to the actual testing, sufficient practice runs were performed in order to prepare participants for each task.

### Data analysis

Descriptive analyses were done for the academic performance indicators and facial affect recognition measures. Individual FAR measures of the Hexagon task were correlated with those of the Animation task using Pearson correlation. FAR measures on both tasks were also individually correlated with the academic performance indicators using Pearson correlation.

The significance levels for the correlations were adjusted to accommodate the many associations of interest; the threshold p-value was set as p = 0.0002 for the Hexagon tasks (n = 237) and as p = 0.002 for the Animation tasks (n = 215). Using these criteria resulted in selecting only those combinations with a Pearson correlation coefficient of 0.2 or above in size, which indicated that the choice of threshold was such that no “spurious” associations were taken forward into the multiple regression analysis. For those passing this threshold, mixed model linear regressions were performed to measure the strength of the associations, adjusting for the covariates; gender and age.

The year effect was incorporated into the model as a random effect, since we were not interested in this effect per se. Loess analysis was first completed to investigate the appearance of the associations, which showed non-linear relationships with the hexagon measures, and were therefore categorized into quartiles for further analysis.

### Ethical approval

Permission to access student examination marks was granted by the Research Ethics Committee of the Faculty of Medicine and Health Sciences, University of Stellenbosch. All participants provided written informed consent and all information was regarded as confidential. This study also received ethical approval from the Committee for Human Research of the University of Stellenbosch.

### Details of funding

The authors hereby acknowledge funding received from the Fund for Innovation and Research into Teaching and Learning, University of Stellenbosch.

## Results

### Demographic data

Two hundred and thirty seven students (n = 237) provided data for the Hexagon task and two hundred and fifteen (n = 215) for the Animation task. Of the 237 students, 21% were from 2008, 70% from 2009, and 9% from 2010. The study group consisted of 144 (61%) female and 93 (39%) male participants, with a mean (SD) age of 24.1 ± 1.6, range 22–35 years.

There were 22 cases where data could not be used for the Animation task due to technical issues. The gender distribution for these 22 cases did not differ from the rest of the cases, but they were slightly older (mean age 25.3 years versus 24.0 years; p = 0.0001) and had a higher average examination mark (66.7% versus 62.2%; p = 0.023).

### Examination scores

The average examination marks ranged between 61%-68% (Table 1). The average total examination mark did not differ between the groups with and without facial affect recognition measurements, supporting the assumption of no bias in the selection of the student group with FAR data.

### Facial affect recognition

In the Hexagon task; happiness was the easiest emotion to identify, disgust and anger were the two most challenging emotions to identify, with the largest variability for measuring disgust (Table 2). In the Animation task; participants were equally able to recognize the three emotions angry, happy, and sad, with the largest variability for measuring happiness (Table 2).

**Table 2:** **Mean percentage (SD) scores for variables on the hexagon task (n = 237) and animation task (n = 215) for medical students at Stellenbosch University**

	**Hexagon task**			**Facial affect recognition task**		
	**Mean%**	**SD**	**CV**	**Mean%**	**SD**	**CV**
Surprise	.89	.12	.1	-	-	
Happy	.98	.06	.1	60.5	16.5	.3
Disgust	.77	.23	.3	-	-	
Fear	.83	.17	.2	-	-	
Sad	.92	.10	.1	58.1	11.4	.2
Anger	.79	.18	.2	62.0	12.9	.2

SD: standard deviation; CV: coefficient of variation - variation in the measurements relative to the mean.

### Association between facial affect recognition and examination scores

Pearson correlation on the individual facial expression recognition measures between the Hexagon and Animation tasks was weak (below .2). For the Hexagon task, there was a significant association between perceptual discrimination of anger and anaesthesiology (*r* = .24; *p* = .004) as well as urology examination scores (*r* = .24; *p* = .001) (Table 3). For the Animation task, there was a significant negative correlation between perceptual discrimination of happiness and O&G examination scores (*r* = −.21, *p* = .002) (Table 3). When gender stratified, there was a significant association between perceptual discrimination of anger and urology for males (*r* = .34; *p* = .02).

**Table 3:** **Correlation between variables on the hexagon and animation tasks and exit examination scores of medical students at Stellenbosch University**

**Correlation (r)**												
	**Surgery**	**Internal medicine**	**Family medicine**	**Paediatrics**	**Psychiatry**	**Obstetrics**	**Anaesthesiology**	**Ear nose and throat**	**Orthopaedics**	**Oncology**	**Urology**	**Mean performance**
**Hexagon task**												
**Anger**	.18	.14	.18	.14	.08	.13	.24**	.14	.03	.11	.24**	.12
**Surprise**	.12	.12	.13	.1	.05	-.01	.09	.09	.04	.09	.10	.03
**Happy**	-.01	-.02	-.03	-.04	-.02	-.05	0^$^	.09	-.04	0^$^	.04	-.06
**Disgust**	0^$^	0^$^	-.04	-.06	-.07	-.02	.01	.04	-.01	.02	.01	-.08
**Fear**	.06	.13	.03	.03	-.05	.11	.05	.07	-.02	.04	.08	.08
**Sad**	.02	.09	-.01	-.1	-.06	-.05	-.02	.05	-.07	-.01	-.01	-.01
**Animation task**												
**Happy**	-.11	-.03	-.12	-.09	-.11	**-.21** ^*^	-.15	.06	-.05	-.03	.01	-.13
**Angry**	-.05	-.03	-.07	0^$^	-.06	-.17	-.1	.06	-.01	-.07	.07	-.09
**Sad**	-.03	-.02	-.04	.02	-.05	-.09	-.01	-.01	-.02	-.02	.01	-.08

^$^r < 0.005; *P = 0.008; **p = 0.0002; Pearson correlations.

### Linear regression analyses

The Loess results indicated that the relationship between anesthesiology scores and Hexagon anger scores was possibly not linear and therefore the quartiles for anger (<0.67, 0.67-0.83, 0.83-0.92, >0.92) were used in further linear regression analysis. The global test (*p* = .003) indicated a significant relationship between anaesthesiology performance and the Hexagon anger score quartiles (adjusted for gender and age). Results showed that the first quartile differed significantly from the second, third and fourth quartile. There was, on average, an increase of 7 marks from the first to the fourth quartile (Table 4). Gender and age were not significant covariates in the model (*p* < .05).

**Table 4:** **Linear regression results for the association between hexagon anger (Quartiles) and anaesthesiology scores of medical students at Stellenbosch University**

**Anger**	**Range of values**	**Mean estimate of Anesthesiology mark**	**SE**	**t**	**p**
Quartile 1	<.67	60.6	2.2		
Quartile 2	.67-.83	65.1	2.2	2.42	.016
Quartile 3	.83-.92	65.3	2.3	2.50	.013
Quartile 4	>.92	67.7	2.2	3.75	.0002

SE: standard error; p value based on the t test and indicates which quartile differs significantly from the baseline value (quartile 1).

**Figure 1:**
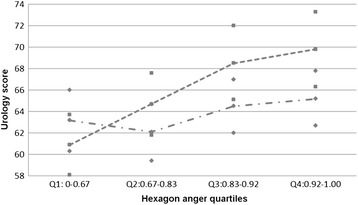
**Linear regression results for the association between urology and hexagon anger recognition scores stratified by gender.** The average marks and the 95% confidence limits are shown. The figure shows that the marks for quartile 1 and 2 are lower than the marks for quartile 3 and 4 for males (top line). There is no difference in marks over the four quartiles for females (bottom line).

The Loess results indicated that the relationship between urology scores and Hexagon anger scores was not linear and therefore the quartiles for anger were used in further linear regression analysis. The global test (*p* = .0001) indicated a significant relationship between urology performance and the Hexagon anger score quartiles. Gender was an effect modifier in the relationship (*p* = .03), with a strong positive relationship for males, but a non-significant relationship for females (Figure 1). Results for males showed that there is an increase in urology marks from the first to the fourth quartile, with an average increase of .9 marks from the first to the fourth quartile (Figure 1). Age was not a significant covariate in the model. In the mixed model of perceptual discrimination of happiness and O&G examination scores (quartiles) the overall test is not significant (p = 0.273).

## Discussion

The association between facial affect recognition, a proposed marker for emotional intelligence, and academic performance was limited in this study to three subjects and three of the emotions, with no overall correlation. The authors know of no prior research on the relationship between FAR and academic performance in medical students. The emotional intelligence (EI) literature shows conflicting associations; some found a positive association between EI and examination performance on a specific course component [28,29] while others show no association between EI and overall academic performance in undergraduate medical students [30]. The field would benefit from further study to deconstruct the concept of EI further. For example, to examine how facial affect recognition influences performance of particular tasks involved in interpersonal interaction.

### Contribution to EI

The study of EI in medical students has been criticized for its broad scope, subjective measures and many confounders (9). Nevertheless, EI continues to be viewed as an important feature of medical doctor success and thus is relevant to undergraduate training (2, 6–8). As is the trend in psychiatry in general, we attempted to move away from the study of this broad concept or phenotype and to examine a more objective and narrower aspect of EI, namely FAR. Possibly even narrower targets are needed in order to find more robust associations between aspects of EI in medical student assessment. Our study shows that there are some associations with academic performance, requiring further study to explain the associations fully.

### Overall non-correlation

The overall non-correlation between FAR and academic performance may be due to the considerable conceptual and methodological complexity inherent in assessing emotional intelligence [31]. The manner in which an individual perceives emotions may be highly functional for him / her and we cannot have certainty that it is “incorrect” purely because of performance in an in vitro task [2]. Test-based understanding does not necessarily correlate with aptitude in life [2]. Nevertheless, these tests are well validated as markers of emotional intelligence and an advantage of our study was that we used an objective measure as opposed to self-report. However this field is far from well understood and much research remains to be done.

Another explanation for the lack of overall correlation is that EI does not influence academic performance independently but rather academic assessment in certain medical schools purposefully or incidentally includes assessment of EI skills. Many of the facets of EI overlap with traditional measures of intelligence [22] and in the field of medicine where interpersonal interaction sometimes forms part of examination this is even more relevant. As medical schools may differ in this respect, this would provide an explanation for the conflicting results in prior literature. Multi-center studies would thus be most useful. Furthermore, if FAR is a marker for EI then the assessment process at each institution and whether they explicitly assess for EI skills or not would have an influence on whether or not FAR and academic performance are associated. Studies such as ours usually explore whether academic performance will be affected by EI but perhaps the more pertinent question is a closer examination of assessment methods and the manner in which EI affects performance in assessments.

### Subject (medical course component or specialty) specific findings

We considered whether subject (e.g. surgery or obstetrics and gynaecology) specific correlation could reflect the use of different assessment methods. However, the complex mixture of assessment methods in the different subjects makes it unlikely that a single assessment method can be viewed as contributory to the relationship between specific facial affect recognition abilities and academic performance in that subject. For example, the urology examination structure consists of an oral examination and three case discussions. On the surface it seems different from the design Obstetrics and Gynaecology (O&G) follow however the OSPE stations of O&G include oral stations and the OSCE/OSPE stations also include case discussion stations. To ascribe the findings to differences in assessment methods only is going to be difficult. Further research is needed to clarify the role of other possible confounding factors. Confounders may include academic structure within a department (i.e. the attitude of teaching personnel and approach to students that might illicit a more fear-based response in students) or the subject matter (e.g. risk of mortality linked to procedures such anaesthesiology would increase the chance of trap-door questions where an incorrect answer on only one key question linked to mortality could lead to failure).

There is little research on the recognition of specific emotions correlated with performance in specific academic subjects. Students who are attracted to a career in fields of urology and anaesthetics or O&G would perhaps work harder and perform better in these subjects due to a combination of natural aptitude, increased interest and harder work ethic for these subjects. Thus we consider below the personality type attracted to these fields in order to hypothesise on the reasons for our subject specific findings.

We found the recognition of anger to be better in students scoring higher in urology. In a study on personality, comparing urologists with other surgeons and non-surgeon doctors; urologists obtained significantly higher extraversion scores than other surgeons and non-surgeons [32]. Of the extraversion facets studied, urologists obtained significantly higher scores on gregariousness and excitement seeking [32]. These personality traits are in keeping with a person who would expose themselves to higher risk situations and who would enjoy social interaction. If one considers this as a genetic trait with an evolutionary advantage – this would be the person in a community who would be at the forefront of interpersonal interactions which may lead to conflict, as opposed to a personality type that withdraws from social interactions for fear of conflict/negative emotions. The former personality type would only thrive if they were able to avoid harm to themselves and this would require a quick apprehension of anger in another person in order to respond quickly to conflict.

Looking at the results together, urology and anaesthetics were both positively correlated with anger while obstetrics was negatively correlated with happiness. Urology and anaesthetics share the surgical realm where control and order and attention to detail are paramount. O&G on the other hand also includes the labour ward as a major focus of training and an environment of relative unpredictability. Although our sample were medical students and not urologists or anaesthetists; one can hypothesize about the different personality type attracted to these different environments and how this may be reflected in emotional intelligence and facial effect recognition and our subject specific findings.

### Gender

The gender effect we found is in keeping with other studies, which showed that males are more accurate than females in recognizing the facial expression of anger [15,33,34]. We did not find overall gender correlation with the facial affect recognition tasks but only correlation with one specific emotion on one task. Some studies have shown gender differences in overall emotional intelligence measures. In a review by Arora et al. [2], 6 out of 7 studies showed females have higher EI scores than males. Hojat et al. [35] showed that gender is associated with empathy and that there was no correlation with objective academic assessments but clinical competence was rated higher by staff members in those with higher empathy scores.

If overall FAR is not correlated with gender as suggested by our study and measures of EI are associated with gender as suggested elsewhere in the literature – could this reflect the social bias introduced in self report measures of EI whereas this is less likely in an objective test of FAR which tests a lower order facet of EI? That is, response bias in EI tests which are self report, may include more gender based biases while FAR tasks which are objective may minimize this. There is some argument that EI is socially constructed and self-report measures would reflect self-representation according to socially desirable norms (particularly relevant in gender) [9].

### Use of these two FAR tasks

We chose two tasks to assess facial affect recognition as they assess partially distinguishable abilities in keeping with their supposed association with different neurological networks (27). The Animation task does not involve labeling of emotions and is less cognitively challenging (25). Our results showed different patterns of association between the tasks and academic performance. We propose that this is because they are indeed testing different underlying capabilities. The Hexagon task showed an association between urology and anaesthesiology performance and anger. The Animation task, which involves only recognizing the emotion, showed an association with obstetrics and happiness. Further study is needed to explain these differences further.

### Limitations

This study was limited by its restriction to final year medical students at a single South African university. There is a possible selection bias for the Animation task that could have biased the results in either direction, but it is unlikely since less than 10% of the Animation task data is missing. All investigated relationships were based on univariate analyses, not taking other confounders (e.g. cognitive abilities) into account. Nevertheless, while we did not consider the full array of skills that comprise emotional intelligence, we examined facial affect recognition; a critical and primary component of EI. We also did not use self-report screening tools with checklist items, since self-ratings measure self- perceptions of emotional skills rather than the skills themselves [34].

## Conclusion

This study furthers the exploration of emotional intelligence by examining facial affect recognition, a proposed building block of EI. EI is of particular interest in the education of medical doctors but FAR has not yet been studied in this group. We found an association between FAR abilities (in detecting happiness and anger) and academic performance measures in specific subjects (obstetrics, urology and anaesthesiology).

The lack of overall correlation between FAR and academic performance may be due to inherent complexities of studying emotional intelligence. Subject specific correlation may reflect aspects of the personality type attracted to certain subjects. Assessment procedures differ between subjects however no direct influence of assessment type was evident due to the multiple and overlapping assessments in each subject. Gender was an effect modifier in the association between facial anger recognition abilities and urology examination marks. Although an association has been found between EI and gender elsewhere there was a lack of overall correlation between gender and FAR in our study. This may reflect that FAR is a more objective marker and a more stable trait while EI is more likely to be influenced by societal factors, which will affect self-report measures and gender particularly.

In keeping with the many aspects of this study, which lack precedent, this study asks many more questions than it answers. This is the nature of the field and should not dissuade further critical exploration.

## Abbreviations

FAR: Facial affect recognition
EI: Emotional intelligence
